# Impact of Ostomy on Quality of Life in Patients with Colorectal Cancer: A Systematic Review and Meta-Analysis

**DOI:** 10.3390/healthcare14040444

**Published:** 2026-02-10

**Authors:** Cristina Díaz-Sánchez, Pedro Manuel Rodríguez-Muñoz, Víctor Navarro-López, Juan Manuel Carmona-Torres, Alba Sánchez-Gil, Juan Luis Sánchez-González, Cristina Rivera-Picón

**Affiliations:** 1 Faculty of Physiotherapy and Nursing, University of Castilla-La Mancha, 45071 Toledo, Spain; cristina.diaz19@alu.uclm.es (C.D.-S.); juanmanuel.carmona@uclm.es (J.M.C.-T.); 2 Department of Physical Therapy, Occupational Therapy, Rehabilitation and Physical Medicine, Rey Juan Carlos University, 28922 Madrid, Spain; victor.navarro@urjc.es; 3 National Centre of Reference for People with Alzheimer’s Disease and Other Dementias, 37008 Salamanca, Spain; asanchezzg02@gmail.com; 4 Department of Medicine, Faculty of Medicine, University of Salamanca, 37007 Salamanca, Spain; juanluissanchez@usal.es; 5 Institute of Biomedical Research of Salamanca (IBSAL), 37007 Salamanca, Spain; 6 Faculty of Physiotherapy and Nursing, University of Castilla-La Mancha, 45600 Talavera de la Reina, Spain; cristinarivera@uclm.es

**Keywords:** quality of life, colorectal cancer, ostomy

## Abstract

**Highlights:**

**What are the main findings?**

- Ostomized patients with colorectal cancer have significantly lower quality of life compared to non-ostomized patients (pooled effect size = −0.29).
- The negative impact of ostomy is consistent across study designs and follow-up periods, confirming the robustness of the findings.

**What are the implications of the main findings?**

- Healthcare professionals should provide tailored psychosocial and rehabilitative support to improve adaptation and emotional well-being in ostomized patients.
- Future research should explore targeted interventions to enhance social reintegration and long-term quality of life in this population.

**Abstract:**

**Background/Objectives**: This systematic review and meta-analysis evaluate the impact of ostomy on quality of life in patients with colorectal cancer, synthesizing evidence from case–control and interventional studies, and to assess the consistency, robustness, and clinical implications of the findings. **Methods**: A systematic search of PubMed, Web of Science, Scopus, and the Cochrane Library was conducted for studies published up to 21 September 2025. Eligibility criteria encompassed cases–control studies comparing ostomized versus non-ostomized colorectal cancer patients and interventional studies assessing quality of life before and after ostomy creation. Data extraction included study design, population characteristics, quality of life outcomes, and main findings. **Results**: A total of 5841 records were identified, with 71 full-text articles assessed and six studies meeting inclusion criteria. The random-effects meta-analysis using the REML estimator yielded a pooled effect size of −0.29 (95% CI: −0.38 to −0.20; *p* < 0.001), indicating significantly lower quality of life in ostomized patients. No heterogeneity was observed (τ^2^ = 0; I^2^ = 7%). Quality assessment indicated that 66.7% of studies were high quality and 33.3% intermediate. Sensitivity analyses confirmed robustness, as exclusion of influential studies did not alter results. **Conclusions**: Ostomy significantly reduces quality of life in colorectal cancer patients. Psychosocial and emotional challenges, including body image concerns and social limitations, contribute to this impact. These findings emphasize the need for comprehensive care and targeted interventions to enhance adaptation, social reintegration, and overall well-being in ostomized patients.

## 1. Introduction

Colorectal cancer (CRC) represents a major global public health challenge due to its high incidence and mortality worldwide. It is currently the third most commonly diagnosed cancer and the second leading cause of cancer-related death globally [1]. In 2020 alone, approximately 1.93 million new CRC cases were diagnosed worldwide, accounting for nearly 10% of all cancer diagnoses [2]. According to World Health Organization (WHO) estimates, cancer ranks as the first or second leading cause of death before the age of 70 years in 112 of 183 countries and as the third or fourth leading cause in an additional 23 countries [3]. CRC incidence demonstrates substantial geographical variation, with similar distribution patterns observed in both men and women [4]. Notably, a rising incidence of CRC among younger individuals (under 50 years of age) has emerged as a concerning global trend [5].

Advances in CRC screening programs and therapeutic strategies have led to marked improvements in survival outcomes, particularly when disease is detected at an early stage. The five-year relative survival rate for stage I CRC is approximately 90%, whereas it declines dramatically to around 14% for stage IV disease [2]. These improvements are largely attributed to earlier diagnosis through population-based screening initiatives, as well as advancements in surgical techniques and adjuvant therapies. Despite these advances, colorectal cancer continues to represent a significant clinical burden, especially among patients who require complex surgical interventions.

Surgical treatment remains the cornerstone of CRC management, especially for early-stage and locally advanced disease. Although substantial progress has been made in chemotherapy, radiotherapy, immunotherapy, and targeted therapies, surgery continues to offer the most effective curative approach for many patients. Over time, CRC surgery has evolved from conventional open techniques to minimally invasive and precision-based approaches, aiming to improve oncological outcomes while reducing postoperative morbidity [6]. However, many patients’ undergoing CRC surgery require the formation of a stoma, either temporarily or permanently, as part of their treatment.

Colostomies and ileostomies are common and standardized surgical procedures in patients diagnosed with CRC. The word stoma or ostomy is derived from the Latin word *ostium*, which means “opening” or “mouth”. An ileostomy or colostomy is created by bringing the small or large bowel, respectively, through the anterior abdominal wall [7]. Intestinal stoma formation is a common surgical procedure in the management of colorectal cancer, particularly in cases requiring low anterior resection or abdominoperineal resection.

Although often life-saving, these procedures are associated with considerable morbidity and substantial changes in bowel function, patients frequently report fatigue, cognitive difficulties, and substantial impairments in daily functioning, all of which contribute to a marked decline in quality of life (QoL) [8,9].

The presence of an ostomy can result in significant physical, psychological, and social challenges for patients. Common ostomy-related complications include skin irritation (76%), pouch leakage (62%), offensive odor (59%), reduced participation in pleasurable activities (54%), and symptoms of depression or anxiety (53%) [10]. As a result, the quality of life of ostomized patients is often substantially compromised across multiple dimensions, including stress, self-esteem, communication, stigma, and body image. Consequently, these individuals require not only ongoing professional healthcare support but also strong family and social support systems to facilitate adaptation and coping [11].

A growing body of evidence has examined quality of life, including health-related quality of life (HRQoL) among CRC patients living with a stoma. Previous research consistently indicates that ostomized patients experience lower HRQoL, with both physical and psychosocial domains being adversely affected. Systematic reviews and recent studies have highlighted declines in physical and functional capacities following CRC treatment [12], as well as the negative impact of ostomy-related problems on perceived quality of life, particularly among patients with colostomies [13]. Furthermore, a 2022 systematic review concluded that stomas are negatively associated with physical function and quality of life in older adults [14], while a 2025 study reported significantly higher levels of body image concerns among patients with an ostomy compared to those without [15]. Importantly, evidence suggests that targeted interventions, including self-care education and psychological support, may mitigate some of these negative effects and improve patients’ quality of life [16]. Additionally, younger ostomates and female patients appear to be more adversely affected in terms of HRQoL [17].

Although previous studies have consistently reported reduced quality of life, including health-related quality of life in patients with an ostomy, the existing evidence remains fragmented and heterogeneous. In particular, prior research lacks quantitative synthesis and shows substantial variability in study design, outcome measures, and populations. Therefore, a systematic synthesis is necessary to quantify the magnitude and consistency of quality of life impairment, examine sources of heterogeneity, and assess differences according to age and stoma type, as well as identify the quality of life domains most affected. Moreover, psychological outcomes are often underreported or inconsistently assessed quality of life.

Despite the growing literature on the impact of ostomies in colorectal cancer (CRC) patients, important knowledge gaps remain. Many studies are limited by small sample sizes, cross-sectional designs, or insufficient consideration of age, stoma type, and cultural context. To date, no focused meta-analysis has simultaneously evaluated quality of life and psychological outcomes specifically in CRC patients with ostomies. Therefore, the objective of this study is to conduct a systematic review and meta-analysis to quantitatively evaluate the impact of ostomies on quality of life and psychological outcomes in patients diagnosed with colorectal cancer, to assess the magnitude and consistency of these effects, and to identify methodological and clinical gaps that may inform future research and the development of tailored supportive interventions.

## 2. Methods

We conducted the systematic review and meta-analysis following Cochrane guidance and reported it in accordance with PRISMA [18] (see Supplementary Tables S1 and S2), MOOSE [19], and the EQUATOR Network guidelines [20]. The protocol is recorded on PROSPERO (CRD420251173538).

### 2.1. Literature Search Strategy

A comprehensive literature search was conducted in PubMed, Web of Science, Scopus, and the Cochrane Library for studies published up to 21 September 2025, with no restriction on the starting year. The search strategy combined the terms (ostom*) AND (“quality of life” OR “life quality” OR “living standard” OR “living conditions” OR “life standards” OR “standard of living”).

Only studies conducted in humans and published in English, Spanish, or Portuguese were considered. Duplicate records were removed using Covidence, and the remaining studies were screened for eligibility using Rayyan.

The search strategy was intentionally broad to maximize sensitivity. Specific relevance to colorectal cancer was ensured during title and abstract screening by including only studies involving ostomized patients due to colorectal cancer and excluding those related to other conditions.

The search strategy was independently reviewed and verified by three reviewers to ensure completeness and accuracy. Full details of the search strategy are provided in the online supplement “Explicit Search Entry” (Supplementary Method S1).

### 2.2. Eligibility Criteria

The inclusion criteria and description of studies in this review followed the PICOS [21] (Population, Intervention, Control, Outcomes, and Study design) framework for reviews:

1. Study design:
  1. Case–control studies comparing patients with colorectal cancer and a stoma (cases) versus patients with colorectal cancer without a stoma (controls), with assessment of quality of life.
  2. Experimental or interventional studies in which patients with colorectal cancer undergo ostomy creation, with quality of life evaluated both before and after the intervention.
2. Population: human adults (≥18 years) diagnosed with colorectal cancer.
3. Outcome measure: assessed health-related quality of life or related patient-reported outcomes.
4. Intervention/Exposure: Surgical creation of a stoma (colostomy or ileostomy) as part of the treatment for colorectal cancer. The intervention group included patients who underwent stoma formation, either temporary or permanent, while the comparison group comprised patients who received colorectal surgery without stoma creation.

Exclusion criteria: studies were excluded if they:

1. Included pediatric populations (<18 years).
2. Were case reports, letters, editorials, reviews, or conference abstracts.
3. Focused exclusively on non-human subjects.
4. Did not provide sufficient data on quality of life or related outcomes.

### 2.3. Study Selection and Data Collection

An initial search retrieved 5841 articles. However, only 6 studies were finally included in the meta-analysis.

After removal of duplicates using Covidence, titles and abstracts were screened independently by two reviewers using Rayyan. Studies meeting the inclusion criteria were assessed in full text to confirm eligibility.

Data extraction was performed independently by two reviewers (Juan Luis Sánchez González and Cristina Díaz Sánchez) using a standardized data collection form. Extracted data included study characteristics (author, year), study design (case–control or interventional), population characteristics, sample size, middle age of the patients, ostomy status, type of ostomy, quality of life outcomes (instruments used and main results), and other relevant findings. Discrepancies were resolved by consensus among reviewers.

### 2.4. Primary Outcome and Effect Size

The primary outcome was health-related quality of life (HRQoL), assessed using validated instruments such as the Short Form Health Survey (SF-36), European Organisation for Research and Treatment of Cancer Quality of Life Questionnaire–Core 30 (EORTC QLQ–C30), World Health Organization Quality of Life–BREF (WHOQOL–BREF), or disease-specific questionnaires.

### 2.5. Risk of Bias and Quality Assessments

Risk of bias for included studies was independently assessed by two reviewers using the ROBINS-E tool for non-randomized studies [22], evaluating domains such as confounding, selection of participants, classification of interventions, deviations from intended interventions, missing data, outcome measurement, and selective reporting. Discrepancies were resolved through discussion or consultation with a third reviewer. Studies were categorized as having low, moderate, or high risk of bias.

The overall methodological quality of observational studies was assessed using the Newcastle-Ottawa Scale (NOS) [23], focusing on selection, comparability, and outcome domains. Quality assessments were used to interpret findings and, when appropriate, to guide sensitivity analyses.

### 2.6. Quality Data Synthesis

Data from included studies were synthesized both narratively and, where appropriate, quantitatively through meta-analysis. Results from individual studies were tabulated in summary tables to facilitate comparison across study designs and quality of life domains. When quantitative synthesis was feasible, meta-analytic results were visually presented using appropriate graphical displays. Studies were grouped according to study design (case–control or interventional), and relevant quality of life domains.

### 2.7. Quantitative Data Synthesis and Analysis

Quantitative data from included studies were synthesized through meta-analysis when sufficient comparable data were available. Continuous outcomes, specifically health-related quality of life scores, were analyzed using mean differences (MD) or standardized mean differences (SMD) with 95% confidence intervals (CI). In principle, if multiple follow-up time points or subgroups were reported in sufficient studies, these could be analyzed separately; however, due to the small number of included studies, subgroup analyses were not performed.

The meta-analysis was performed using Review Manager statistical software (version 5.4; Cochrane, London, UK) and the metafor package in R (version 4.4.0; R Foundation for Statistical Computing, Vienna, Austria). Effect sizes (SMDs) were calculated based on means and standard deviations (SDs) extracted from each study. When studies reported medians with interquartile ranges, these were converted into means and SDs. When standard errors were reported, they were transformed into SDs. If numerical data were unavailable, corresponding authors were contacted to obtain the results; otherwise, data were estimated from figures using ImageJ software (version 1.54p; National Institutes of Health, Bethesda, MD, USA). Studies lacking sufficient information were excluded from the quantitative synthesis and described narratively.

The meta-analysis employed the inverse variance method with a random-effects model and 95% confidence intervals. The choice of a random-effects model was established a priori and was not determined post hoc based on observed heterogeneity. This approach yields more conservative estimates when clinical heterogeneity is expected, considering differences in populations, interventions, and measurement timings. Assessment of publication bias was not performed because fewer than 10 studies were included, in accordance with methodological recommendations that consider such analyses unreliable in small meta-analyses. Statistical significance was set at *p* < 0.05, and effect sizes (SMDs) of ≥0.8 were classified as large, 0.5–0.8 as moderate, and 0.2–0.5 as small.

Heterogeneity among studies was assessed using the Chi^2^ test (Cochran’s Q) and quantified with the I^2^ statistic, with thresholds of 25%, 50%, and 75% indicating low, moderate, and high heterogeneity, respectively. Due to the limited number of studies included per outcome, no subgroup analyses or meta-regression were performed to explore potential sources of heterogeneity. Sensitivity analysis was conducted using a leave-one-out approach to assess the influence of individual studies on the overall pooled estimate and ensure robustness of results. The certainty of the evidence for each outcome was assessed using the GRADE approach, considering risk of bias, inconsistency, indirectness, imprecision, and potential publication bias.

## 3. Results

### 3.1. Study Selection and Characteristics

**Figure 1:**
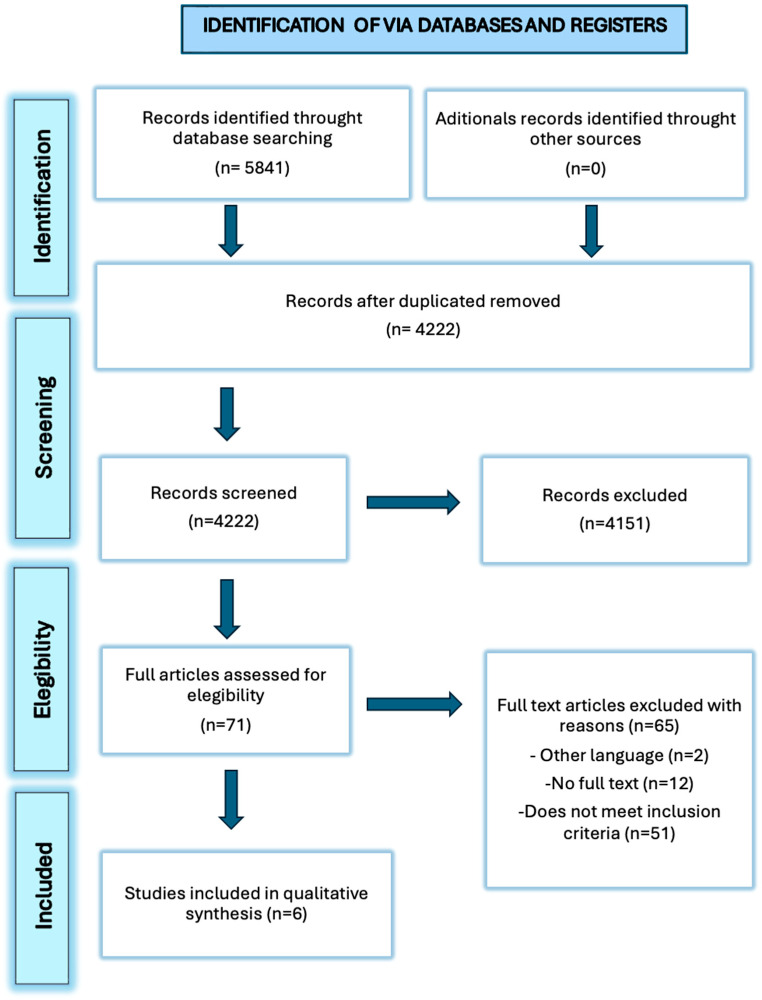
Flowchart.

Our literature search identified 5841 studies across the selected databases. No additional records were retrieved from other sources. After removing duplicates, 4222 articles were screened by title and author, of which 4151 were excluded for not meeting the inclusion criteria based on title and abstract screening resulting in 71 articles selected for full-text review evaluated for eligibility. Of these, 65 articles were excluded for the following reasons: publication in a non-eligible language (n = 2), lack of access to the full text (n = 12), or failure to meet the predefined inclusion criteria (n = 51). Finally, 6 studies met the inclusion criteria and were included in the review (Figure 1).

As a result, 5 studies were cases and controls studies (83.4%) and 1 study (16.6%) was an experimental study. The characteristics of the studies are detailed in Table 1.

The sample sizes ranged from 48 to 2299 participants. The variables most frequently evaluated included quality of life, psychiatric morbidity, stress, psychiatric well-being, cope, resilience, depression, anxiety, comorbidity and perception of the illness Study populations mainly consisted of adult patients, with or without ostomy. In several studies, participants were classified into two groups: ostomized and non-ostomized patients [24,25,26,27,28,29].

Across the five cases–control studies included, data from 4250 patients diagnosed with colorectal cancer were analyzed. Of these, 1332 were ostomized patients (cases), and 2918 were non-ostomized patients (controls) [24,25,26,27,28].

However, in the experimental study, Carlsson et al. [29], analyzed 57 individuals with rectal cancer and an ostomy, of whom 47 had a colostomy and 13 had an ileostomy. Quality of life and worries were assessed before surgery and at 1, 3, and 6 months postoperatively, and the results were compared with a control group with 22 patients [29].

Overall, the six studies included in this review consistently highlight the multidimensional impact of having an ostomy among colorectal cancer survivors, with quality of life emerging as the most frequently assessed outcome. Although some studies reported minimal or non-significant differences between ostomized and non-ostomized patients, others identified substantial impairments—particularly in younger individuals and in domains related to physical, role, and social functioning. Evidence also shows that psychological aspects, including stress, coping, psychiatric morbidity, illness perception, and specific ostomy-related concerns, contribute meaningfully to patient well-being. Experimental longitudinal data further demonstrate that HRQOL declines markedly during the first postoperative month but progressively improves over time despite persistent concerns such as fatigue, pain, and fear of recurrence. Collectively, these findings indicate that the presence of an ostomy can influence quality of life and psychological adjustment in diverse ways, underscoring the importance of tailored postoperative support and comprehensive assessment of both physical and psychosocial outcomes [24,25,26,27,28,29].

### 3.2. Qualitative Results of Quality of Life

Across the six included studies, quality of life was assessed using both generic and disease-specific instruments, with several studies providing detailed qualitative insights into the lived experience of colorectal cancer survivors with and without ostomies.

Three studies used the EORTC QLQ-C30, with or without the disease-specific QLQ-CR38: Verweij et al. [24] and Mols et al. [28] reported that ostomized patients experienced significantly lower physical, role, and social functioning, as well as reduced global health status. Notably, Mols et al. [28] observed additional concerns regarding body image and male sexual functioning, while ostomized patients paradoxically reported fewer gastrointestinal symptoms compared to non-ostomized participants. These findings highlight persistent functional and psychosocial challenges in daily life after ostomy surgery.

Two studies applied SF-36 measures: Carlsson et al. [29] reported an initial drop in physical and role functioning shortly after colorectal surgery, with partial recovery by six months, whereas Mohler et al. [26] emphasized long-term deficits in physical, social, and psychological wellbeing among ostomy patients. Both studies underscored the lasting impact of surgical interventions on daily functioning and self-perceived health.

Michelone et al. [25] employed the WHOQOL-bref and found no statistically significant differences in overall QoL between ostomized and non-ostomized survivors. However, ostomized participants consistently scored slightly lower across physical, psychological, social, and environmental domains, suggesting subtle but meaningful QoL impacts not captured by statistical tests alone.

Simpson et al. [27] provided a more nuanced, qualitative perspective, showing that stoma-related complications, particularly leakage frequency, strongly influenced multiple dimensions of QoL, including physical, mental, social, and environmental wellbeing. Participants described coping strategies such as diet regulation, checking the stoma bag, and wearing loose clothing, reflecting the daily challenges and self-management burden associated with living with a stoma.

In summary, qualitative insights from these studies indicate that ostomy presence and complications are consistently associated with lower physical and social functioning, altered body image, and psychosocial challenges. While disease-specific instruments like the EORTC QLQ-C30/CR38 and ostomy-specific questionnaires capture clinically relevant deficits, generic measures such as SF-36 and WHOQOL-brief provide broader contextual understanding of survivors’ adaptation and wellbeing. Overall, these findings suggest that individualized support and interventions addressing both physical and psychosocial domains are critical for improving QoL in ostomy patients [24,25,26,27,28,29].

### 3.3. Effect Size

**Figure 2:**
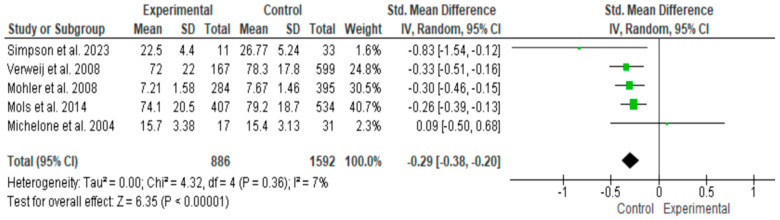
Forest plot showing the standardized mean differences (Hedges’ g) and 95% confidence intervals for each included study, as well as the overall pooled estimate. Negative values indicate poorer quality of life in the ostomy group compared to the non-ostomy group [24,25,26,27,28].

The random-effects meta-analysis using the restricted maximum likelihood (REML) estimator yielded a pooled effect size of −0.29 (95% CI: −0.38 to −0.20; *p* < 0.001), indicating a significant difference between groups, with higher quality of life observed in the control group compared to the ostomy group. No heterogeneity was detected (τ^2^ = 0; I^2^ = 7%; Q = 4.32, *p* = 0.36), suggesting high consistency across studies (Figure 2).

### 3.4. Quality Assessment

Overall, the quality of the studies ranged from 3 to 9 in the Newcastle–Ottawa Scale, with a median of 7 and a mean of 6.92 (s.d. = 1.78). 0 studies (0%) were rated as low quality (score < 6); 2 (33.3%) as intermediate quality (scores 6–7) and 4 (66.6%) obtained a high-quality score (Supplementary Table S3).

### 3.5. Risk of Bias

**Figure 3:**
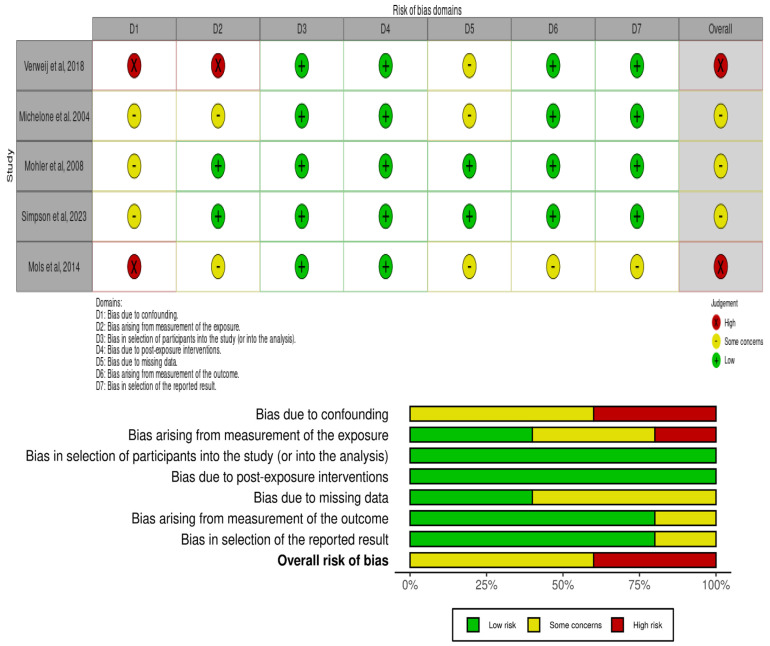
Risk of bias [24,25,26,27,28].

In the Figure 3, we can observe that two studies (40%) [24,28] were high risk; and three (60%) [25,26,27] had some risk of bias. The ROBINS-E D1 item (confounding bias) was the independent item with the highest risk of bias.

### 3.6. Sensitivity Analysis

The influence analysis identified one study with slightly greater statistical weight (Mols et al., 2014 [28]), mainly due to its large sample size. However, omitting any single study did not substantially affect the pooled estimate. The leave-one-out sensitivity analysis showed that pooled estimates ranged narrowly between −0.29 and −0.31, with I^2^ = 0% in all cases. These findings confirm the robustness and stability of the overall effect, indicating that no single study disproportionately influenced the results.

### 3.7. Certainty of the Evidence

The certainty of the evidence for health-related quality of life outcomes was assessed using the GRADE approach and rated as moderate–low. Risk of bias: Two studies (40%) were judged as high risk of bias, and three studies (60%) had some risk of bias. The ROBINS-E D1 item (confounding bias) was the domain with the highest risk. Inconsistency: Heterogeneity was low (I^2^ = 0%), and leave-one-out sensitivity analyses showed that omitting any single study did not substantially affect the pooled estimate, indicating consistent results across studies. Imprecision: The pooled effect size was −0.29 (95% CI: −0.38 to −0.20), showing a precise estimate. Indirectness: All studies directly assessed health-related quality of life in ostomy versus control populations, with no concerns regarding indirectness.

Overall, these assessments indicate moderate–low certainty that individuals with an ostomy have lower quality of life than control participants.

## 4. Discussion

This systematic review and meta-analysis examined the impact of ostomy formation on quality of life and psychological outcomes in colorectal cancer survivors, synthesizing evidence from six studies with heterogeneous designs, populations, and outcome measures. Overall, the findings consistently indicate that ostomy formation is associated with reduced health-related quality of life, although the magnitude and affected domains vary across patient groups and study methodologies [24,25,26,27,28,29]. This impact is multidimensional, involving physical, psychological, social, and illness-perception components, and often persists long-term, highlighting the complex challenges faced by survivors living with a stoma [15,30,31,32,33,34,35,36,37,38,39,40].

Physical limitations—such as fatigue, reduced mobility, difficulties with daily and work-related activities, and stoma management challenges—were consistently reported [13,26,36,41]. These issues not only impose a direct physical burden but also contribute to emotional distress, social withdrawal, and decreased confidence in self-management. Psychological and social factors, including body image disturbances, sexual dysfunction, and illness perceptions, further influenced survivors’ well-being [13,14,15,27,28,29,30,32,36,37]. Emotional distress, including anxiety and depressive symptoms, was widely reported and could persist beyond the immediate postoperative period [27,29,30,34,37,38]. Caregivers also experienced substantial emotional strain, impacting patient well-being through reciprocal family dynamics [35,39]. Self-care competence consistently emerged as a key determinant of HRQoL, with effective self-management predicting better outcomes [33,34], whereas inadequate training or difficulties with the stoma apparatus were associated with poorer outcomes [13,26,36,41].

Demographic and contextual factors modulated HRQoL. Younger survivors experienced more pronounced disruptions to body image, identity, and social functioning [24,34], and women often reported lower HRQoL than men [13]. Social support consistently acted as a protective factor, facilitating adaptation and buffering against emotional distress [34,35,39]. These findings emphasize the need for tailored interventions that consider age, gender, social context, and individual coping resources.

Despite the robustness of these findings, several limitations should be noted. The included studies were heterogeneous in design, populations, outcomes, and follow-up duration, which may limit comparability. Some studies relied on self-reported outcomes, introducing potential bias. The small number of studies precluded subgroup analyses by demographic or clinical variables. Methodological variability in measurement tools, especially ostomy-specific outcomes, highlights the need for standardized assessments in future research [42].

This systematic review and meta-analysis demonstrated that the presence of a stoma is associated with a statistically significant reduction in health-related quality of life. The pooled standardized mean difference (−0.29; 95% CI: −0.38 to −0.20; *p* < 0.001) indicates a small-to-moderate negative effect in favor of the non-ostomy groups, with a striking absence of heterogeneity (I^2^ = 0%). Sensitivity analyses further confirmed the robustness of these findings, as the pooled effect remained stable in all leave-one-out iterations. These results align with existing evidence showing that patients living with a stoma often experience multidimensional burdens affecting physical, emotional, social, and spiritual well-being [42,43]. Narrative syntheses have reported similar trends, pointing to common challenges such as impaired body image, social withdrawal, and emotional distress [42]. Importantly, modifiable factors—such as adequate stoma site marking, patient education, physical activity, and sustained family or social support—have been highlighted as potentially protective influences [33,44,45].

Taken together, our results contribute robust quantitative evidence indicating that patients with a stoma experience a measurable reduction in HRQoL. However, the comparison with external studies reveals important clinical implications: structured continuing-care programs, patient education, and interventions that enhance self-care can mitigate this decline [33,44,45]; demographic and anatomical factors may identify vulnerable subgroups requiring targeted support [13,46]; and qualitative insights emphasize the importance of individualized psychological and social adaptation [42,43]. Future research should integrate standardized HRQoL measures with utility-based outcomes, conduct well-designed prospective studies, and evaluate long-term intervention effects. Ultimately, improving HRQoL in ostomy patients demands a holistic approach that combines medical, educational, psychosocial, and rehabilitative strategies.

The results have important implications for practice, policy, and research. Comprehensive, multidisciplinary support programs—including self-management training, psychosocial interventions, and ongoing education—are critical for improving HRQoL [33,44,45]. Younger survivors, those experiencing body image distress, or socially isolated individuals may require targeted support. Policy initiatives could include structured post-discharge care programs and long-term follow-up services [45]. Future research should adopt standardized HRQoL measures, evaluate long-term outcomes, and investigate targeted interventions to optimize adaptation and well-being [42,43,46,47].

In conclusion, this review demonstrates that ostomy formation in colorectal cancer survivors has a measurable, multidimensional, and persistent impact on quality of life and psychological well-being [24,25,26,27,28,29,42,43]. Addressing this burden requires a holistic approach integrating medical, educational, psychosocial, and rehabilitative strategies tailored to the complex needs of this patient population.

### 4.1. Strengths and Limitations

This review presents several strengths. First, it was conducted in accordance with the PRISMA guidelines and was prospectively registered in PROSPERO, ensuring methodological transparency and reducing the risk of reporting bias. In addition, a comprehensive literature search was performed across multiple databases, which increases the likelihood of having captured the most relevant and up-to-date evidence on the topic.

Although this review provides meaningful insights, several limitations should be acknowledged. Only six studies met the inclusion criteria, which reduces statistical power and constrains the generalizability of the findings. Most of the included studies were observational in nature; therefore, their conclusions may be influenced by unmeasured confounding factors and do not allow causal relationships to be established. Additionally, none of the studies reported sex-stratified analyses, preventing the exploration of potential gender differences in stoma perception and its impact on quality of life. Some studies reported multiple follow-up time points, but the limited number of studies prevented analysis of changes over time or subgroup analyses by follow-up duration. Moreover, the scarcity of available research, the heterogeneity of the measurement instruments employed, and the inherent risk of confounding further limit the robustness and consistency of the conclusions drawn from this review. Future studies using standardized tools, larger and more diverse populations, and more rigorous methodological approaches are needed to strengthen the evidence base. One limitation of this study is that the population included individuals with ostomies of different etiologies, it may limit the generalizability of the findings to specific subgroups, such as patients with ostomies due to colorectal cancer.

### 4.2. Clinical Implications

Overall, integrating quantitative and qualitative evidence underscores the need for comprehensive care strategies that address both the physical and psychosocial aspects of living with an ostomy. Healthcare professionals should implement interventions that promote emotional well-being, social reintegration, caregiver support, and culturally sensitive guidance. Future research should investigate longitudinal outcomes and evaluate the effectiveness of structured programs aimed at mitigating the multifaceted challenges associated with ostomy and enhancing overall quality of life.

## 5. Conclusions

This systematic review and meta-analysis indicates that colorectal cancer survivors with an ostomy experience a small-to-moderate but consistent reduction in health-related quality of life. The most affected dimensions were physical functioning, role and social participation, body image, and emotional well-being. Younger individuals and those facing stoma-related complications appear particularly vulnerable. These findings underscore the need for targeted postoperative support integrating physical rehabilitation, psychosocial care, and stoma self-management training. Future research should prioritize robust longitudinal designs to evaluate tailored interventions aimed at improving adaptation and long-term quality of life.

## Figures and Tables

**Table 1:** Characteristics of studies included.

Study	Study Design	Population (n)	Groups (n)	Ostomy Status	Age (Year)	Outcomes	Results
Verweij et al. (2018) [24]	Case–control study	CRC patients (2299)	Case group (494)	Ostomy patients	60.2	Quality of life (EORTC QLQ-C30 and EORTC QLQ-C38)	Elderly CRC patients with an ostomy report more limitations in physical functioning compared without an ostomy.
			Control group (1805)	No Ostomy patients	60.4		
Michelone et al. (2004) [25]	Case–control study	CRC patients (48)	Case group (17)	Ostomy patients	66.5	Quality of life (WHOQOL)	No significant differences were found in the quality of life between ostomates and non-ostomates.
			Control group (31)	No Ostomy patients	60.9		
Mohler et al. (2008) [26]	Case–control study	CRC patients (679)	Case group (284)	Ostomy patients	72.4	Quality of life (mCOH-QOL ostomy and SF-36 V2)	The quantitative measures used were acceptable. Mixed methods design allows for assessment of long-term QOL.
			Control group (395)	No Ostomy patients	71.1		
Simpson et al. (2023) [27]	Case–control study	CRC patients (278)	Case group (129)	Ostomy patients	46	Quality of life (WHOQOL-BREF) Psychiatric morbidity (GHQ-12) Psychiatric well-being (WEMWBS) Stress (PSS) Cope (Brief Coping Scale) Resilience (CD-RISC).	Psychological well-being and general physical health are reduced in stomates.
			Control group (149)	No Ostomy patients	33		
Mols et al. (2014) [28]	Case–control study	CRC patients (946)	Case group (408)	Ostomy patients	69.8	Quality of life (EORTC QLQ-C30 and EORTC QLQ-C38) Depression and Anxiety (HADS) Comorbidity (SCQ) Perception of the illness (BIPQ)	Survivors with colorectal cancer and stoma have lower QOL and worse illness perceptions than those without stoma 1 to 10 years after diagnosis.
			Control group (538)	No Ostomy patients	67.9		
Carlsson et al. (2010) [29]	Intervention	Rectal Cancer (57)	Ostomy patients (57)	Colostomy (44) Ileostomy(13)	66	Quality of life (SF-36) Worries (RFIPC)	Surgical management of rectal cancer raises concerns and profoundly impairs QOL during the first several postoperative months.
			Control group (22)	No ostomy patients	-		

CRC = Colorectal Cancer. EORTC QLQ-C30 = European Organisation for Research and Treatment of Cancer Quality of Life Questionnaire–Core 30. EORTC QLQ-C38 = European Organisation for Research and Treatment of Cancer Quality of Life Questionnaire—Core 38. WHOQOL = World Health Organization Quality of Life. mCOH-QOL ostomy = modified City of Hope Quality of Life of Ostomates. SF-36 = 36–item Short Form Health Survey. SF-36 V2 = 36–item Short Form Health Survey, version 2. GHQ-12 = General Health Questionnaire 12–items. WEMWBS = Warwick–Edinburgh Mental Well-being Scale, WHOQOL-BREF: World Health Organization Quality of Life–BREF. PSS = Perceived Stress Scale. CD-RISC = Connor-Davidson Resilience Scale. HADS = Hospital anxiety and depression scale. QOL = Quality of life. SCQ = Self-administered Comorbidity Questionnaire. BIPQ = Brief Illness Perception Questionnaire. RFIPC = Rating Form of Inflammatory Bowel Disease Patient Concerns.

## Data Availability

Not applicable.

## Supplementary Materials

The following supporting information can be downloaded at: https://www.mdpi.com/article/10.3390/healthcare14040444/s1.

## Abbreviations

The following abbreviations are used in this manuscript:

BIPQ	Brief Illness Perception Questionnaire
CD-RISC	Connor-Davidson Resilience Scale
CI	Confidence Interval
CRC	Colorectal Cancer
EORTC QLQ-C30	European Organisation for Research and Treatment of Cancer Quality of Life Questionnaire–Core 30
EORTC QLQ-C38	European Organisation for Research and Treatment of Cancer Quality of Life Questionnaire–Core 38
GHQ-12	General Health Questionnaire–12 items
HADS	Hospital Anxiety and Depression Scale
HRQoL	Health-Related Quality of Life
MD	Mean Difference
mCOH–QOL	Modified City of Hope–Quality of Life
NOS	Newcastle–Ottawa Scale
PSS	Perceived Stress Scale
QoL	Quality of Life
RFIPC	Rating Form of Inflammatory Bowel Disease Patients’ Concerns
REML	Restricted Maximum Likelihood
SCQ	Self-administered Comorbidity Questionnaire
SD	Standard Deviation
SE	Standard Error
SF-36	Short Form Health Survey
SF-36 V2	Short Form Health Survey, version 2
SMD	Standardized Mean Difference
UK	United Kingdom
USA	United States of America
WEMWBS	Warwick–Edinburgh Mental Well-Being Scale
WHO	World Health Organization
WHOQOL-BREF	World Health Organization Quality of Life–BREF
