# Supplementary material for: Impact of Ostomy on Quality of Life in Patients with Colorectal Cancer: A Systematic Review and Meta-Analysis
Source: Healthcare (Basel). 2026 Feb 10;14(4):444. doi: 10.3390/healthcare14040444 (PMC12941021; doi:10.3390/healthcare14040444)
Supplement: Supplementary file 1 [file healthcare-14-00444-s001.zip › Supplementary eTable1.pdf]

Table 1.

Study	Study Design	Population (n)	Groups (n)	Ostomy status	Age (Years)	Outcomes	Results
**Verweij et al. (2018)**	Case control study	CRC patients (2299)	Case group (494)	Ostomy patients	60.2	Quality of life (EORTC QLQ-C30 and EORTC QLQ-C38)	Elderly CRC patients with an ostomy report more limitations in physical functioning compared with their counterparts without an ostomy.
			Control group (1805)	No Ostomy patients	60.4		
**Michelone et al. (2004)**	Case control study	CRC patients (48)	Case group (17)	Ostomy patients	66.5	Quality of life (WHOQOL)	No significant differences were found in the quality of life between ostomates and non-ostomates.
			Control group (31)	No Ostomy patients	60.9		
**Mohler et al. (2008)**	Case control study	CRC patients (679)	Case group (284)	Ostomy patients	72.4	Quality of life (mCOH-QOL ostomy and SF-36 V2)	The quantitative measures used were acceptable. Mixed methods design allows for assessment of long-term QOL.
			Control group (395)	No Ostomy patients	71.1		
**Simpson et al. (2023)**	Case control study	CRC patients (278)	Case group (129)	Ostomy patients	46	Quality of life (WHOQOL-BREF)<br>Psychiatric morbidity (GHQ-12)<br>Psychiatric well-being (WEMWBS)<br>Stress (PSS)<br>Cope (Brief Coping Scale)<br>Resilience (CD- RISC).	Phycological well-being and general physical health are reduced in stomates.
			Control group (149)	No Ostomy patients	33		
**Mols et al. (2014)**	Case control study	CRC patients (946)	Case group (408)	Ostomy patients	69.8	Quality of life (EORTC QLQ-C30 and EORTC QLQ-C38)<br>Depression and Anxiety (HADS)<br>Comorbidity (SCQ)<br>Perception of the illness (BIPQ)	Survivors with colorectal cancer and stoma have lower QOL and worse illness perceptions than those without stoma 1 to 10 years after diagnosis.
			Control group (538)	No Ostomy patients	67.9		
**Carlsson et al. (2010)**	Intervention	Rectal Cancer (57)	Ostomy patients (57)	Colostomy (44)<br>Ileostomy(13)	66	Quality of life (SF-36)<br>Worries (RFIPC)	Surgical management of rectal cancer raises concerns and profoundly impairs QOL during the first several postoperative months.
			Control group (22)	No ostomy patients			

Characteristics of studies included

CRC= Colorectal Cancer. **EORTC-QLQ-C30**= European Organization for Research and Treatment of Cancer Quality of Life Questionnaire. **EORTC QLQ-C38**= European Organization for Research and Treatment of Cancer Quality of Life Questionnaire of patients with colorectal cancer. **WHOQOL=** The World Health Organization Quality of Life. **mCOH-QOL ostomy**= The modified City of Hope Quality of Life of Ostomates. **SF 36=** The 36-item Short Form Health Survey. **SF-36 V2**= The 36-item Short Form Health Survey version 2. **GHQ-12=** General Health Questionnaire 12-items. **WEMWBS=** The

Warwick-Edinburgh Mental Well-being Scale, **WHOQOL-BREF**: The World Health Organization–abridged version. **PSS=**Perceived Stress Scale. **CD-RISC=** Connor-Davidson Resilience Scale. **HADS=** Hospital anxiety and depression scale. **QOL=** Quality of life. **SCQ=** Self-administered Comorbidity Questionnaire. **BIPQ=** Brief Illness Perception Questionnaire. **RFIPC=** The Rating Form of Inflammatory Bowel Disease Patient Concerns.
