# Supplementary material for: Impact of Ostomy on Quality of Life in Patients with Colorectal Cancer: A Systematic Review and Meta-Analysis
Source: Healthcare (Basel). 2026 Feb 10;14(4):444. doi: 10.3390/healthcare14040444 (PMC12941021; doi:10.3390/healthcare14040444)
Supplement: Supplementary file 1 [file healthcare-14-00444-s001.zip › Supplementary eTable3.pdf]

Supplementary Table 3 Quality evaluation results of NOS included in the study.

Study	Selection				Comparability	Outcome			Total score*
	Representativeness of the exposed cohort	Selection of the non exposed cohort	Ascertainment of exposure	Demonstration that outcome of interest was not present at start of study	Comparability of cohorts on the basis of the design or analysis	Assessment of outcome	Was follow-up long enough for outcomes to occur	Adequacy of follow up of cohorts	
Verweij et al. (2018)	*	*	*	*	**	*	*	*	9/9
Michelone et al. (2004)	*	*	*	*	*	*	X	X	7/9
Mohler et al. (2008)	*	*	*	*	**	*	*	*	9/9
Simpson et al. (2023)	*	*	*	*	**	*	*	*	9/9
Mols et al. (2014)	*	*	*	*	**	*	*	*	9/9
Carlsson et al. (2010)	*	X	*	*	X	*	*	*	7/9

*: Maximum score is 9. Scores <6 were classified as low-quality studies, 6 to 7 as intermediate-quality studies, and 8 to 9 as high-quality studies.
