# Supplementary material for: Impact of Ostomy on Quality of Life in Patients with Colorectal Cancer: A Systematic Review and Meta-Analysis
Source: Healthcare (Basel). 2026 Feb 10;14(4):444. doi: 10.3390/healthcare14040444 (PMC12941021; doi:10.3390/healthcare14040444)
Supplement: Supplementary file 1 [file healthcare-14-00444-s001.zip › Supplementary Method S1.pdf]

<u>Suplementary eMethods: Database formulas during literatura search</u>
<u>Actualizado 21/9</u>

<u>PubMed Search Formula: 3127</u>
(ostom*) AND (“quality of life” OR “life quality” OR “living standard” OR “living conditions” OR “life standards” OR “standard of living”)

<u>Cochrane Library Search Formula: 627</u>
((ostom*) AND (“quality of life” OR “life quality” OR “living standard” OR “living conditions” OR “life standards” OR “standard of living”))

<u>SCOPUS Search Formula: 1298</u>
TITLE-ABS-KEY (ostom*) AND (“quality of life” OR “life quality” OR “living standard” OR “living conditions” OR “life standards” OR “standard of living”)

<u>WOS Search Formula: 789</u>
(ostom*) (Topic) AND (“quality of life” OR “life quality” OR “living standard” OR “living conditions” OR “life standards” OR “standard of living”) (Topic)

<u>Criterios de inclusión/exclusión. Tipos de estudio a seleccionar</u>

<u>Criterios de inclusión:</u>
1. Estudios transversales de casos y controles.
2. Estudios experimentales con intervención, en los que hay un grupo control y un grupo intervención.

<u>Criterios de exclusión:</u>
El resto de los estudios no comprendidos en los mencionados anteriormente.
